# The Value of Cerebral Blood Volume Derived from Dynamic Susceptibility Contrast Perfusion MRI in Predicting IDH Mutation Status of Brain Gliomas—A Systematic Review and Meta-Analysis

**DOI:** 10.3390/diagnostics15070896

**Published:** 2025-04-01

**Authors:** José Pablo Martínez Barbero, Francisco Javier Pérez García, Paula María Jiménez Gutiérrez, Marta García Cerezo, David López Cornejo, Gonzalo Olivares Granados, José Manuel Benítez, Antonio Jesús Láinez Ramos-Bossini

**Affiliations:** 1 Advanced Medical Imaging Group (TeCe22), Instituto de Investigación Biosanitaria de Granada (ibs.GRANADA), 18012 Granada, Spain; josep.martinez.sspa@juntadeandalucia.es (J.P.M.B.); wajaviray@gmail.com (F.J.P.G.); paulajg@correo.ugr.es (P.M.J.G.); martagarcia_99@outlook.com (M.G.C.); davidlc@correo.ugr.es (D.L.C.); j.m.benitez@decsai.ugr.es (J.M.B.); 2 Department of Radiology, Hospital Universitario Virgen de las Nieves, 18014 Granada, Spain; 3 Department of Anesthesiology, Hospital Universitario Virgen de las Nieves, 18014 Granada, Spain; 4 Centro de Genómica e Investigación Oncológica (GENYO), 18016 Granada, Spain; 5 Department of Computer Science and Artificial Intelligence, University of Granada, 18071 Granada, Spain; 6 Department of Neurosurgery, Hospital Universitario Virgen de las Nieves, 18014 Granada, Spain; gonzalo.olivares@ugr.es; 7 Department of Human Anatomy and Embryology, School of Medicine, University of Granada, 18016 Granada, Spain

**Keywords:** glioma, brain, magnetic resonance imaging, dynamic susceptibility contrast, cerebral blood volume, neuroimaging

## Abstract

**Background:** Dynamic susceptibility contrast perfusion MRI (DSC-MRI) is a promising non-invasive examination to predict histological and molecular characteristics of brain gliomas. However, the diagnostic accuracy of relative cerebral blood volume (rCBV) is heterogeneously reported in the literature. This systematic review and meta-analysis aims to assess the diagnostic accuracy of mean rCBV derived from DSC-MRI in differentiating Isocitrate Dehydrogenase (IDH)-mutant from IDH-wildtype gliomas. **Methods:** A comprehensive literature search was conducted in PubMed, Web of Science, and EMBASE up to January 2025, following PRISMA guidelines. Eligible studies reported mean CBV values in treatment-naïve gliomas with histologically confirmed IDH status. Pooled estimates of standardized mean differences (SMDs), diagnostic odds ratios (DOR), and area under the receiver-operating characteristic curve (AUC) were computed using a random-effects model. Heterogeneity was assessed via I^2^ statistic. Meta-regression analyses were also performed. **Results:** An analysis of 18 studies (*n =* 1733) showed that mean rCBV is significantly lower in IDH-mutant gliomas (SMD = −0.86; *p* < 0.0001). The pooled AUC was 0.80 (95% CI, 0.75–0.90), with moderate sensitivity and specificity. Meta-regression revealed no significant influence of DSC-MRI acquisition parameters, although a flip angle showed a trend toward significance (*p* = 0.055). **Conclusions:** Mean rCBV is a reliable imaging biomarker for IDH mutation status in gliomas, demonstrating good diagnostic performance. However, heterogeneity in acquisition parameters and post-processing methods limits generalizability of results. Future research should focus on standardizing DSC-MRI protocols.

## 1. Introduction

Gliomas are the most common primary malignant brain tumors [1,2], prompting extensive research to improve their characterization. Traditionally, the most relevant factors considered in glioma classification, established by the World Health Organization, were based on histological characteristics. However, a fundamental paradigm shift occurred in the 2016 WHO classification, which emphasized molecular such as Isocitrate Dehydrogenase (IDH) mutation status and 1p/19q codeletion to improve diagnostic accuracy and prognostic stratification of gliomas [3]. This change was consolidated and further extended in the most recent WHO classification (2021) [4]. Therefore, differentiating IDH-mutant from IDH-wild type gliomas is of paramount importance, since these subtypes have distinct prognostic and therapeutic implications

Considering the need for assessing tumor biomarkers in order to establish the WHO grade of a glioma, invasive methods to obtain a histological specimen of the tumor are necessary, with stereotactic biopsy being the most frequent procedure [5]. However, this technique entails patient risks, including intracranial hemorrhage, infection, or neurological deficits [6]. Additionally, small biopsied tissue samples may not fully capture the heterogeneity of gliomas, potentially leading to misclassification or underestimation of aggressive components within the tumor [7]. These limitations have warranted the search for non-invasive imaging techniques capable of providing reliable molecular characterization of brain glioma without the need for tissue sampling.

Magnetic Resonance Imaging (MRI) is an essential examination in glioma assessment, offering insights into tumor morphology, vascularization, and metabolic activity [8]. Conventional MRI sequences, including T1-weighted, T2-weighted, and FLAIR imaging, are widely used for glioma detection and anatomical delineation. However, these sequences provide limited information regarding tumor biology as they only offer structural information [9,10]. Advanced MRI techniques, such as Diffusion-Weighted Imaging (DWI), Magnetic Resonance Spectroscopy (MRS), and Perfusion-Weighted Imaging (PWI), have gained increasing attention for their ability to assess tumor microstructure, metabolism, and hemodynamics, respectively [10]. Among these, Dynamic Susceptibility Contrast perfusion MRI (DSC-MRI) has demonstrated particular promise in evaluating glioma vascular properties, distinguishing tumor subtypes [11,12], and differentiating progression from pseudoprogression [13].

Cerebral Blood Volume (CBV), derived from DSC-MRI, has been extensively studied as a biomarker of tumor vascularity, particularly as a normalized measure (rCBV) [14]. This perfusion metric is calculated by measuring the area under the curve (AUC) of the signal-intensity–time curve during the first pass of a bolus of gadolinium-based contrast agent, which is usually normalized to the mean value of normal-appearing white matter (NAWM) in the contralateral hemisphere to account for inter-patient variability [15]. In biological terms, rCBV measures the volume of blood within a given amount of brain tissue, reflecting tumor vascularity. Thus, high rCBV values indicate increased tumor angiogenesis, which is a hallmark of high-grade tumors [16].

Accordingly, brain gliomas with higher vascular proliferation, such as IDH-wildtype glioblastomas, exhibit elevated rCBV values, whereas IDH-mutant gliomas, known for their lower angiogenic activity, tend to have lower rCBV values [17]. This biological distinction supports that rCBV may serve as a reliable non-invasive imaging biomarker for predicting IDH mutation status, offering a potential alternative to invasive biopsy. In fact, several studies have reported promising results regarding the utility of rCBV in glioma molecular stratification [18].

However, significant variability exists in reported diagnostic performance, likely due to differences in imaging acquisition parameters, post-processing techniques, or tumor segmentation strategies [19]. Similarly, there is significant variability in the reported specific rCBV values that offer the best diagnostic performance in terms of probabilistic distribution. Mean rCBV, max rCBV, and different specific percentiles of rCBV have been studied, with variable outcomes across studies [20,21]. Overall, this heterogeneity limits the generalizability and consistency of rCBV as a surrogate biomarker for IDH mutation status.

Understanding the impact of technical and methodological variations on rCBV measurements is crucial for standardizing its use in clinical practice [22]. Currently, the most widely used rCBV metric in clinical practice is mean rCBV, which is commonly calculated in an automatic or semi-automatic fashion by most commercial software packages. Although previous works have analyzed the role of different rCBV parameters in differentiating IDH-mutant- vs. IDH-wild-type gliomas, to our knowledge, no previous meta-analyses have been performed specifically aimed at assessing mean rCBV in this diagnostic challenge.

In this study, we performed a comprehensive systematic review and meta-analysis to determine the between-group differences in CBV and the diagnostic accuracy of mean rCBV in distinguishing IDH-mutant from IDH-wildtype gliomas. Additionally, we explored the influence of DSC-MRI acquisition parameters on mean rCBV diagnostic performance. This synthesis will be helpful not only in terms of increasing supporting evidence but also in emphasizing the need for standardization of DSC-MRI performance.

## 2. Materials and Methods

### 2.1. Eligibility Criteria

The design of the meta-analysis and the selection criteria were based on the PICO search strategy. The population was formed by patients with histologically confirmed brain glioma; the intervention was DSC-MRI with derived CBV values; the comparator was the IDH mutation status (mutant vs. wild-type); the primary outcomes were the difference in mean CBV values and the predictive value of mean rCBV. The Preferred Reporting Items for Systematic Reviews and Meta-Analyses (PRISMA) [23] guidelines were followed in the design and writing of the study (the PRISMA checklist can be consulted in Supplementary File S1). The protocol of the study was registered in the Open Science Framework registry [24].

Accordingly, the inclusion criteria were the following: published observational or experimental studies evaluating DSC-MRI-derived CBV values in treatment-naive gliomas with known IDH status. The exclusion criteria were studies not providing mean (r)CBV values or other CBV values permitting a reliable estimation of mean (r)CBV, studies not reporting IDH mutation status, studies solely reporting outcomes of MRI perfusion modalities other than T2*-DSC, and studies focused on machine learning methods that did not provide diagnostic yield metrics of CBV following conventional statistical approaches. In addition, case reports, editorials, and other article formats different from original studies were excluded.

### 2.2. Information Sources and Search Strategy

Two authors (FJPG and DLC) searched the PubMed, Web of Science, and EMBASE databases. Different search equations were carried out, and a final consistent equation was constructed, including combinations derived from a main equation as follows: “(perfusion OR dynamic) AND (magnetic resonance imaging OR MRI) AND (brain OR cerebr* OR “central nervous system”) AND (glioma) AND (gene* OR IDH OR 1p/19q deletion OR MGMT) (the full search strategies in PubMed, Web of Science, and EMBASE can be consulted in Supplementary Files S2, S3 and S4, respectively)”. To increase the sensitivity of the search, a cluster search was performed examining the references of all fully read articles. No language restrictions were established. The search was updated to 1 January 2025.

All titles and abstracts of interest were screened, and those which did not meet the eligibility criteria were excluded. Next, the screened studies were read in full to assess whether they met all eligibility criteria. Discrepancies during the article selection process were solved by a third author (JPMB).

### 2.3. Measured Variables

For each study, the main characteristics were collected, including first author, year, country, study design, sample size (total and in each IDH group), and number of MRI machines involved in the study. In addition, we gathered available information on MRI acquisition parameters (TE, TR, Flip Angle, Slice Thickness, Slice Gap, Matrix, Field of View, contrast agent dose) and post-processing information (commercial software used, arterial input function modality, tumor segmentation modality). The primary outcomes were the mean CBV values for mutated and wild-type IDH and the diagnostic test performance (AUC) of mean rCBV to differentiate between them.

### 2.4. Data Extraction

Two authors (PMJG and FJPG) independently extracted the data from the selected articles, and a third author (AJLRB) reviewed the data and solved any discrepancies. If a study reported data for a subgroup of patients from the main cohort, only data related to patients with known IDH mutation status were collected. All data were annotated in a spreadsheet for ulterior analysis.

### 2.5. Meta-Regression Analyses

To explore the potential moderating influence of DSC-MRI acquisition parameters as a source of heterogeneity in the main primary outcomes, we performed meta-regression analyses, including echo time (TE), repetition time (TR), flip angle (FA), slice thickness (ST) and gap (ST), acquisition time, and number of images per DSC sequence (dynamic images).

### 2.6. Quality and Publication Bias Assessment

The QUADAS-2 tool [25] was used to systematically assess the existence of potential risks of bias and applicability concerns. For each study, two authors (PMJG and FJPG) classified the risk of bias as low, unclear, or high in each of the four risk of bias items and in the three applicability concern items. An overall risk of bias estimation was provided by a consensus. In case of discrepancy, a third author (AJLRB) was consulted. On the other hand, the publication bias was analyzed using funnel plots and Egger’s tests.

### 2.7. Statistical Analysis

To ensure a robust estimation of the overall effect size, we applied a random-effects model using the restricted maximum likelihood (REML) method to estimate standard mean differences (SMD) of mean (r)CBV between IDH-mutant- and IDH-wild-type gliomas. To assess the diagnostic accuracy of rCBV, we performed a hierarchical-summary receiver-operating characteristic (HSROC) analysis complemented with calculation of the pooled Youden J Index (Sensitivity + Specificity − 1), as well as bivariate random-effects meta-analysis for sensitivity and specificity. Additionally, we computed the pooled AUC, diagnostic odds ratio (DOR), and Youden index for each study when available. Importantly, the analyses were performed separately for studies reporting AUC values for continue (r)CBV values or for specific cutoffs.

When a study reported data for a given glioma WHO grade, it was sub-divided into different subsets as if they were individual studies for data analysis. When data were not explicitly provided, but were presented in graphs, extraction of visual data using the free version of PlotDigitizer tool [26] was performed. Moreover, to avoid excluding relevant studies from analysis, when mean (r)CBV values or other essential metrics, such as AUC, were not explicitly reported, they were estimated to obtain reliable data in accordance with the recommendations of the Cochrane Handbook for Systematic Reviews of Interventions [27,28], as follows:

- Estimation of mean and standard deviation

If a study did not provide the mean and standard deviation of (r)CBV, we estimated these values based on the median and IQR using the method proposed by Wan et al. (2014) for asymmetric distributions [29], as follows:

μ=Q2+0.2·(Q3−Q1)

where *μ* is the mean; Q1, Q2, and Q3 are the first, second (i.e., median), and third quartile values of the distribution. Similarly, for the SD (*σ*), the following formula was applied:

$$\sigma =\frac{Q3-Q1}{1.35}$$

Finally, in some studies, mean/SD (r)CBV values were provided for subgroup comparisons involving a third group (e.g., IDH-mutated with and without 1p19q codeletion). In these cases, combined values were also estimated for the IDH-mutated group (merging both subgroups), weighting the means and standard deviations according to their respective sample sizes.

- Estimation of missing AUC values from different sources

In some studies, AUC values were not directly reported but were instead provided for different percentiles or could be inferred from other performance metrics. Since excluding these studies could have entailed an underestimation of the pooled AUC, we applied well-established statistical methods (e.g., Monte Carlo simulations) to estimate AUC values in those studies where they were not explicitly reported. Such estimations allowed us to retain valuable data and minimize the risk of selection bias, improving the robustness of the analyses. Specifically, based on the available information in each study, we applied the following approaches to estimate AUC values:

If a study reported AUC values for different (r)CBV percentiles rather than a single metric of AUC value for mean (r)CBV, we calculated an overall AUC estimate by computing a weighted average of the reported percentiles. The initial weight was assigned assuming that the percentiles represented equal strata of the (r)CBV distribution. This was further refined using a weighting proportional to the total sample size. The standard error of the estimated AUC was derived following the approach of Hanley & McNeil (1982) [30], according to the following formula:

$${SE}_{AUC}=\sqrt{\frac{AUC\;\cdot \left(1-AUC\right)+{(N}_{1}-1)\;\cdot (Q_{1}-{AUC}^{2})+{(N}_{2}-1)\;\cdot (Q_{2}-{AUC}^{2})}{N_{1}\cdot \;N_{2}}}$$

Similarly, a study reported mean (r)CBV values for different percentiles in each IDH subgroup but did not provide the AUC corresponding to the mean, limiting itself to indicating that this metric did not differentiate (*p* > 0.05) between the mutated- and wild-type IDH groups. To estimate the AUC of the mean rCBV, a simulation method based on Monte Carlo was used, which is widely used in diagnostic accuracy studies when individual data are not available and only summary statistics are available [31,32].

First, normal distributions were modeled for each group using the reported mean and SD values, under the assumption that rCBV follows an approximately normal distribution within each group. Based on these distributions, simulated values of rCBV were generated for each group according to their respective sample sizes, ensuring that the variability within each group was consistent with the reported SD. Subsequently, this simulated sample was used to calculate the receiver-operating characteristics (ROCs) curve and obtain the estimated AUC. To evaluate the uncertainty associated with this estimate, a bootstrap resampling analysis was performed (1000 iterations), obtaining a 95% confidence interval (95% CI). In addition, the optimum cut-off point for the mean rCBV was determined using the Youden index.

Finally, when only the odds ratio or β coefficient values were reported, an estimation of the AUC was made based on the coefficient β obtained in the univariate logistic regression. To do this, the relationship between the coefficient β and the OR was used, following the method described by Zhou et al. (2002) [33], where the AUC can be calculated from the OR using the formula:

$$AUC=\frac{{OR}^{0.5}}{1+{OR}^{0.5}\;}$$

When AUC 95% CIs were not explicitly provided in a given study, they were estimated based on the standard error (SE) according to the Hanley & McNeil formulae [30], as follows:

$$SE=\frac{\sqrt{AUC\;\cdot (1-AUC)}}{\sqrt{N}}\;\;\;\;\;\;\;\;\;\;\;\;\;\;\;\;\;\;\;\;\;\;\;\;\;\;\;95\%CI=AUC\;\pm 1.96\;\cdot SE$$

- Estimation of other diagnostic performance metrics

Other relevant values assessed in this meta-analysis that were not directly reported in the original study were estimated. For OR, the weighted overall OR was calculated using an adjusted mean of the values provided at each percentile, applying a logarithmic adjustment for the 95% confidence interval. Similarly, when the sensitivity and specificity values were not directly reported, they were calculated from the predictive values collected in the study.

Finally, we applied the I^2^ and tau^2^ statistics to assess heterogeneity among studies with non-relevant, moderate, and considerable cut-off values set at I^2^ < 40%, 40% < I^2^ < 75%, and I^2^ > 75%, respectively [34,35,36,37]. Sensitivity analyses were performed to evaluate the robustness of the results. First, a sensitivity analysis was carried out based on the exclusion of studies with imputed data, comparing the SMD and AUC estimates with and without these studies, evaluating changes in effect size and heterogeneity. In addition, an overall sensitivity analysis was performed using the leave-one-out technique, in which each study was sequentially excluded to determine its individual impact on the pooled estimates. Changes in point estimates and heterogeneity were analyzed to identify potentially influential studies and to assess the stability of the results. To visually display the results, forest plots were generated, showing the effect sizes and 95% CIs for individual studies as well as the overall pooled estimates.

## 3. Results

### 3.1. Search Results and Main Characteristics of the Included Studies

**Figure 1:**
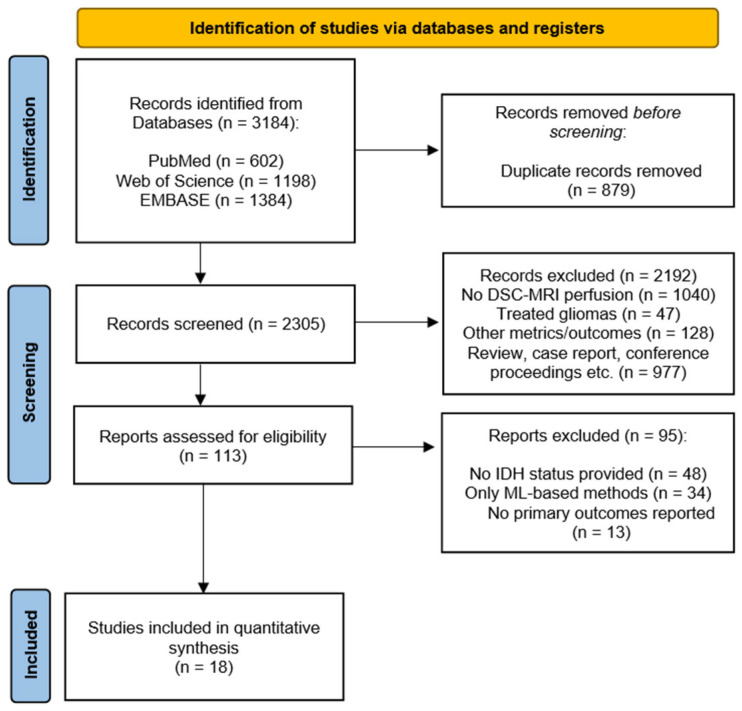
PRISMA flow diagram of the systematic review and meta-analysis.

The initial search in the three databases identified a total of 3184 articles. After duplicate removal (879 articles) and title/abstract screening, 113 articles were fully read. Following the inclusion and exclusion criteria, a total of 18 studies were finally included in the quantitative synthesis. The PRISMA flow chart of the study can be consulted in Figure 1.

The main characteristics of each study are described in Table 1. Further information regarding DSC-MRI post-processing data and MRI acquisition parameters in each study can be consulted in Supplementary Tables S1 and S2, respectively.

### 3.2. Quality Assessment

**Figure 2:**
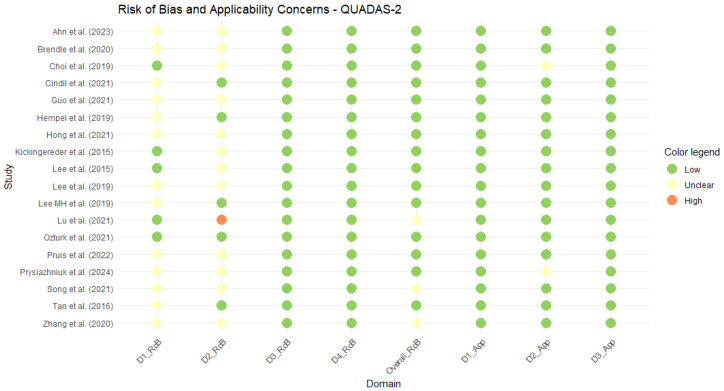
Traffic light plot of the QUADAS-2 assessment for the studies included in the meta-analysis. Ahn et al. (2023) [38], Brendle et al. (2020) [39], Choi et al. (2019) [40], Cindil et al. (2022) [41], Guo et al. (2022) [42], Hempel et al. (2018) [43], Hong et al. (2021) [44], Kickingereder et al. (2015) [45], Lee et al. (2015) [46], Lee et al. (2019) [47], Lee MH et al. (2019) [48], Lu et al. (2021) [49], Ozturk et al. (2021) [50], Pruis et al. (2022) [51], Prysiazhniuk et al. (2024) [52], Song et al. (2020) [53], Tan et al. (2016) [54], Zhang et al. (2020) [55].

The 18 studies included in the meta-analysis showed a low overall risk of bias, except for three [49,53,55] that were categorized as having an unclear risk due to some concerns in patient selection (Figure 2). Further details on the assessment of risk of bias and applicability concerns domains can be found in Supplementary Table S3.

### 3.3. Differences in Mean Cerebral Blood Volume Based on the IDH Status

**Figure 3:**
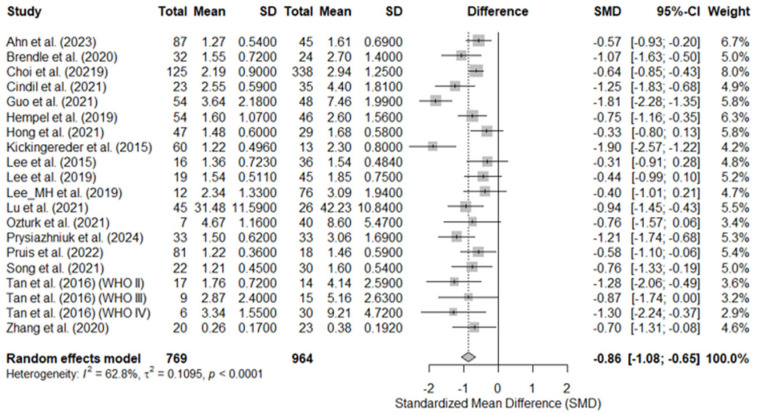
Forest plot for the standard mean difference (SMD) in mean relative cerebral blood volume between IDH-mutated- (left columns) vs. IDH-wild-type brain gliomas (right columns). The horizontal lines indicate the 95% confidence intervals (95% CI). The pooled SMD was −0.86, indicating significantly lower rCBV in IDH-mutant gliomas. WHO II–IV, 2007 World Health Organization central nervous system tumor grades II–IV. Ahn et al. (2023) [38], Brendle et al. (2020) [39], Choi et al. (2019) [40], Cindil et al. (2022) [41], Guo et al. (2022) [42], Hempel et al. (2018) [43], Hong et al. (2021) [44], Kickingereder et al. (2015) [45], Lee et al. (2015) [46], Lee et al. (2019) [47], Lee_MH et al. (2019) [48], Lu et al. (2021) [49], Ozturk et al. (2021) [50], Prysiazhniuk et al. (2024) [52], Pruis et al. (2022) [51], Song et al. (2020) [53], Tan et al. (2016) (WHO II) [54], Tan et al. (2016) (WHO III) [54], Tan et al. (2016) (WHO IV) [54], Zhang et al. (2020) [55].

Statistically significant differences were found in the SMD, being lower in the IDH-mutant group, both overall (SMD = −0.86; *p* < 0.0001) and in each individual study. The greatest between-group difference was found in Kickingereder et al. [45]: (SMD = −1.90), whereas the smallest difference was observed in Lee et al. (2015) [46]: (SMD = −0.30). In five studies [44,46,47,48,50], the 95%CI for SMD included positive SMD values. The overall heterogeneity was moderate (I^2^ = 62.8%; tau^2^ = 0.11), indicating that there are methodological or population differences between the included studies. Figure 3 shows the forest plot of the studies included in the analysis.

### 3.4. Diagnostic Performance of Mean rCBV

#### 3.4.1. Pooled AUC Based on Reported Mean rCBV Cutoff Values

The analysis of the studies that reported an AUC for a specific cut-off value showed a pooled AUC estimate of 0.83 (95% CI: 0.75–0.90), indicating that the mean rCBV value has a good discriminatory capacity for differentiating gliomas with mutated vs. wild-type IDH. However, the heterogeneity between studies was moderate (I^2^ = 68.0%, *p* = 0.0009), suggesting that there are methodological or population differences between the included studies.

**Figure 4:**
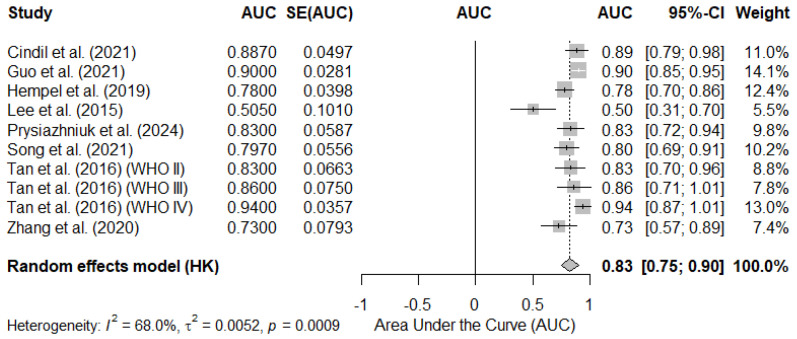
Forest plot of the area under the curve (AUC) in studies reporting a cut-off value for the mean rCBV in the differentiation of gliomas with mutated- vs. wild-type IDH. Each study is represented by a square, the size of which is proportional to the weight of the study in the meta-analysis. The horizontal lines indicate the 95% confidence intervals (95% CIs). The pooled AUC estimate under a random-effects model is represented by the diamond at the bottom of the figure. SE, standard error. Cindil et al. (2022) [41], Guo et al. (2022) [42], Hempel et al. (2018) [43], Lee et al. (2015) [46], Prysiazhniuk et al. (2024) [52], Song et al. (2020) [53], Tan et al. (2016) (WHO II) [54], Tan et al. (2016) (WHO III) [54], Tan et al. (2016) (WHO IV) [54], Zhang et al. (2020) [55].

The individual studies reported AUCs ranging from 0.50 to 0.94, reflecting variability in the selected rCBV thresholds and in the populations studied. Figure 4 shows the forest plot of this analysis.

#### 3.4.2. Pooled AUC in Studies Reporting rCBV as Continuous Values

**Figure 5:**
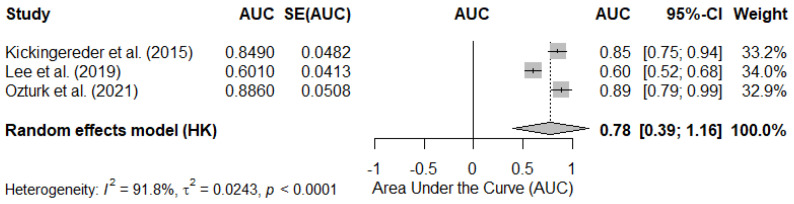
Forest plot of the area under the curve (AUC) in studies reporting the mean rCBV value as a continuous variable to differentiate gliomas with IDH-mutant- vs. IDH-wild-type. Each study is represented by a square, the size of which is proportional to the weight of the study in the meta-analysis. The horizontal lines indicate the 95% confidence intervals (95% CIs). The pooled AUC estimate under a random-effects model is represented by the diamond at the bottom of the figure. SE, standard error. Kickingereder et al. (2015) [45], Lee et al. (2019) [47], Ozturk et al. (2021) [50].

The meta-analysis of the studies that reported an AUC for rCBV as a continuous variable showed a pooled AUC estimate of 0.78 (95% CI: 0.39–1.16). However, the heterogeneity between studies was considerable (I^2^ = 91.8%, *p* < 0.0001). Individual studies reported AUCs ranging from 0.60 to 0.89, indicating significant dispersion in the discriminatory capacity of rCBV when analyzed as a continuous variable. Figure 5 shows the forest plot of this analysis.

#### 3.4.3. Bivariate Meta-Analysis of Sensitivity and Specificity: HSROC Analysis

**Figure 6:**
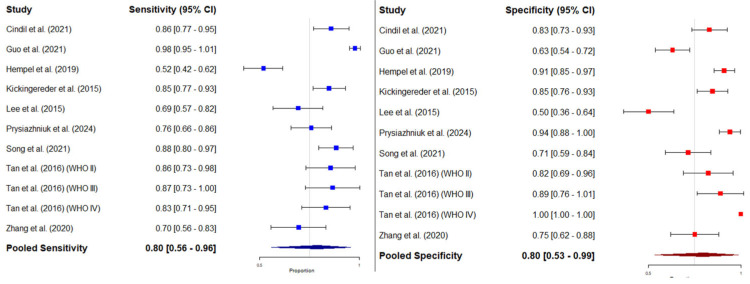
Pooled estimates of sensitivity and specificity for rCBV in predicting IDH mutation status. **Left panel**: Forest plot of sensitivity estimates from individual studies, together with the pooled sensitivity derived from the meta-analysis using a random-effects model. **Right panel:** Forest plot of specificity estimates from individual studies, together with the pooled specificity derived from the meta-analysis using a random-effects model. Each square represents the point estimate of a single study, with horizontal lines indicating the 95% confidence intervals (CI). The combined estimates are shown as diamonds at the bottom of each panel, and their width represents the 95% CI. Cindil et al. (2022) [41], Guo et al. (2022) [42], Hempel et al. (2018) [43], Kickingereder et al. (2015) [45], Lee et al. (2015) [46], Prysiazhniuk et al. (2024) [52], Song et al. (2020) [53], Tan et al. (2016) (WHO II) [54], Tan et al. (2016) (WHO III) [54], Tan et al. (2016) (WHO IV) [54], Zhang et al. (2020) [55].

The forest plots in Figure 6 illustrate the pooled sensitivity and specificity of mean rCBV for distinguishing IDH-mutant- from IDH-wild-type gliomas across multiple studies. Individual sensitivity values were variable, with a pooled sensitivity estimate of 0.80 (95% CI: 0.56–0.96). Similarly, the pooled specificity was 0.80 (95% CI: 0.53–0.99). For individual studies, the ones reporting highest sensitivity and specificity values were those by Guo et al. (2021) [42] and Tan et al. (2016, WHO IV) [54] (0.98 and 1, respectively). Conversely, the studies reporting lowest sensitivity and specificity values were Hempel et al. (2019) [43] and Lee et al. (2015) [46] (0.52 and 0.50, respectively).

**Figure 7:**
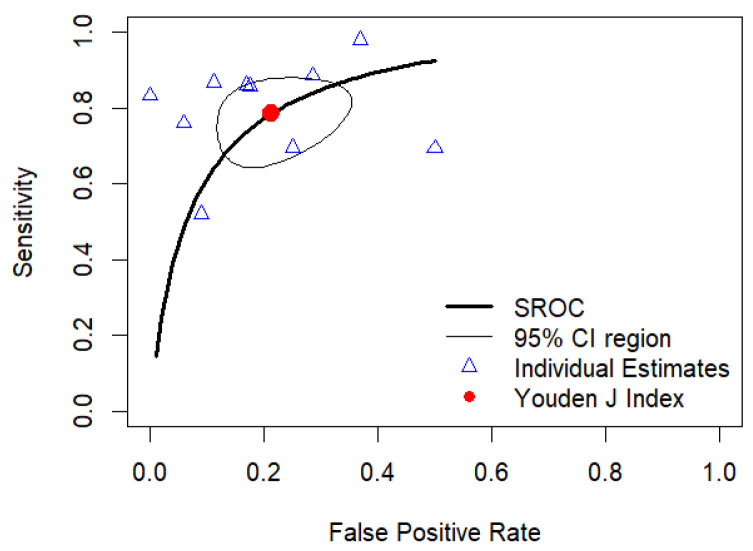
Hierarchical-summary receiver-operating characteristics (HSROC) curve of the diagnostic performance of mean rCBV to classify brain gliomas into IDH-mutant- vs. IDH-wild-type for the studies reporting mean rCBV cutoff values that were included in the meta-analysis.

The results of the HSROC analysis were similar to those of the bivariate meta-analysis, with pooled sensitivity and specificity values of 0.784 (95%CI, 0.674–0.865) and 0.787 (95%CI, 0.678–0.867), respectively, and a pooled AUC of 0.789 (95%CI, 0.779–0.794). Figure 7 shows the HSROC curve of diagnostic performance.

#### 3.4.4. Diagnostic Odds Ratio of Mean rCBV

**Figure 8:**
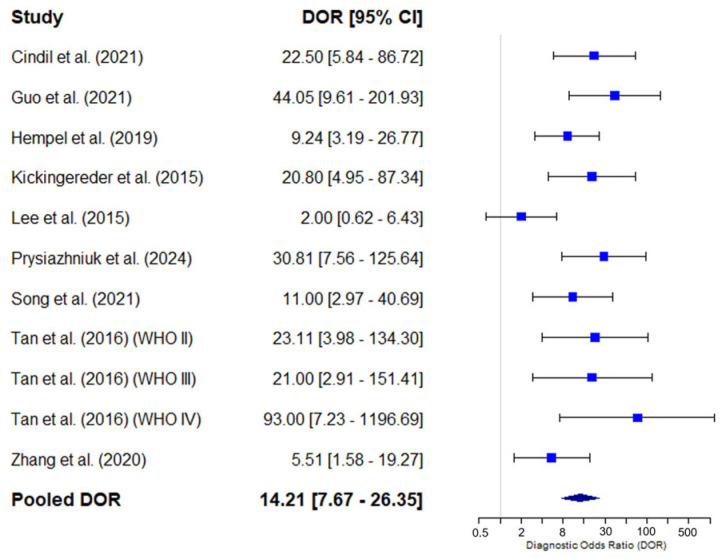
Forest plot showing the diagnostic odds ratio (DOR) and its 95% confidence interval (95% CI) for each individual study included in the meta-analysis. The squares represent the point estimates of the DOR for each study, and the horizontal lines indicate the 95% CIs. The size of the squares is proportional to the weight of each study in the analysis. The blue diamond represents the combined estimate of the pooled DOR using a random-effects model, with its corresponding confidence interval. Cindil et al. (2022) [41], Guo et al. (2022) [42], Hempel et al. (2018) [43], Kickingereder et al. (2015) [45], Lee et al. (2015) [46], Prysiazhniuk et al. (2024) [52], Song et al. (2020) [53], Tan et al. (2016) (WHO II) [54], Tan et al. (2016) (WHO III) [54], Tan et al. (2016) (WHO IV) [54], Zhang et al. (2020) [55].

**Figure 9:**
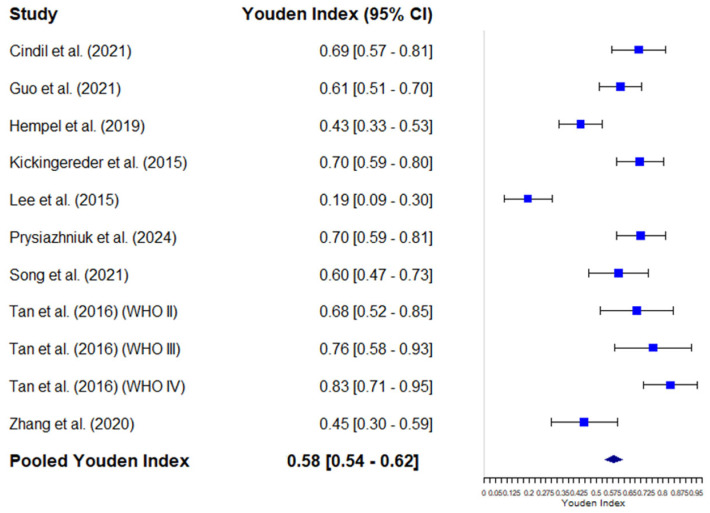
Forest plot of the Youden J index and its 95% confidence interval (95% CI) for each individual study included in the meta-analysis. The squares represent the point estimates of the DOR for each study, and the horizontal lines indicate the 95% CIs. The size of the squares is proportional to the weight of each study in the analysis. The blue diamond represents the combined estimate of the pooled DOR using a random-effects model, with its corresponding confidence interval. Cindil et al. (2022) [41], Guo et al. (2022) [42], Hempel et al. (2018) [43], Lee et al. (2015) [46], Kickingereder et al. (2015) [45], Prysiazhniuk et al. (2024) [52], Song et al. (2020) [53], Tan et al. (2016) (WHO II) [54], Tan et al. (2016) (WHO III) [54], Tan et al. (2016) (WHO IV) [54], Zhang et al. (2020) [55].

The individual results of the studies show considerable variability, with DOR values ranging from 2.00 [0.62–6.43] to 93.00 [7.23–1196.69]. The combined DOR estimate using a random-effects model was 14.21 [7.67–26.35]. The pooled Youden J Index was 0.58, with considerable variability ranging from 0.54 to 0.62. Figure 8 shows the forest plot of the DOR analysis, and Figure 9 shows the forest plot of the Youden J Index of individual and pooled data.

### 3.5. Meta-Regression Analyses

None of the DSC-MRI acquisition parameters showed significant influence in the primary outcomes, but the flip angle showed a trend toward significance (*p* = 0.055) in the SMD meta-regression, with decreasing units being associated with reduced SMD values (β) = −0.009). Heterogeneity was moderate in all cases, ranging from 66.7% to 78.27% in SMD, and from 44.4% to 84.2% in AUC. Table 2 and Table 3 show the results of the meta-regression analyses for SMD and AUC estimates, respectively.

### 3.6. Publication Bias and Sensitivity Analyses

**Figure 10:**
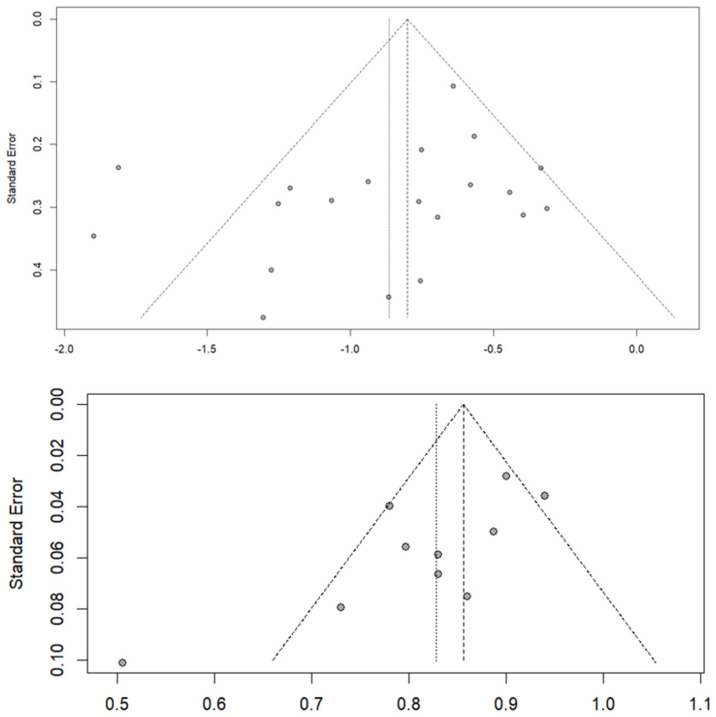
Funnel plots for studies included in the standardize mean difference (**top**) and area under the curve (**bottom**) analyses. The line centered at the vertex of the triangle indicates the estimation according to the random-effects model, and the parallel vertical line on the left refers to the estimation according to a common-effects model.

Regarding publication bias, the shape of the funnel plot for the 20 studies included in the SMD analysis indicated no significant biases, which was supported by the results of the Egger’s test (t = −1.45; *p* = 0.166). The variance of residual heterogeneity (tau^2^ = 2.541) indicated high variability between studies, in agreement with the dispersion observed in the funnel plot. Similar results were observed in the funnel plot of the 10 studies reporting mean rCBV cutoff values that were used to estimate pooled AUC (Egger’s test, t = −2.81; *p* = 0.0227; tau^2^ = 1.765). Figure 10 shows the corresponding funnel plots.

**Figure 11:**
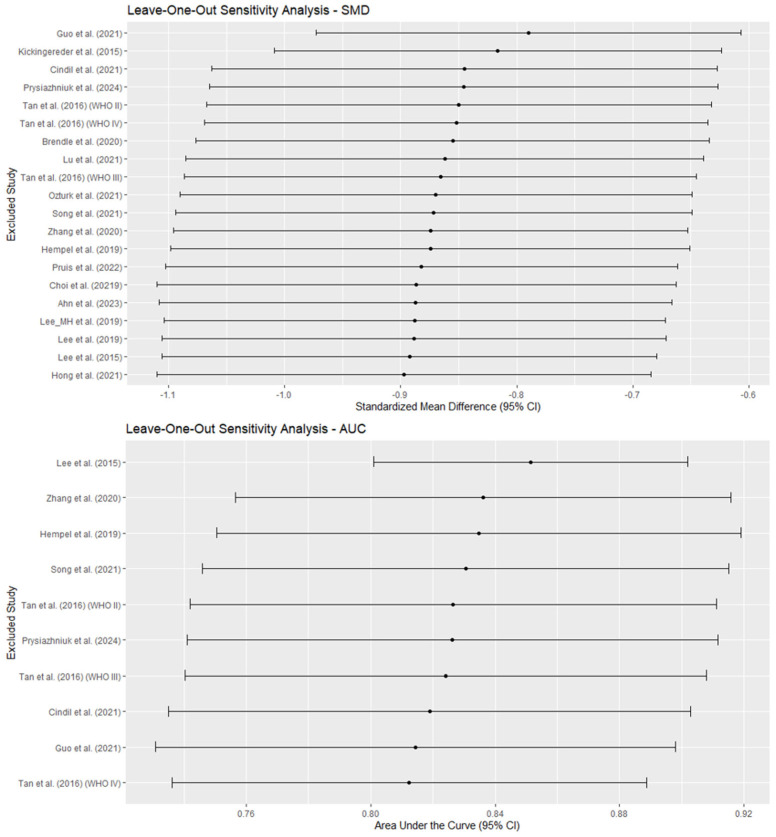
This figure illustrates the results of the leave-one-out sensitivity analysis for the standardized mean difference (SMD) (**top**) and the area under the curve (AUC) (**bottom**). Each point represents the pooled-effect estimate after excluding a single study, with horizontal lines indicating the corresponding 95% confidence intervals (95% CIs). Ahn et al. (2023) [38], Brendle et al. (2020) [39], Choi et al. (2019) [40], Cindil et al. (2022) [41], Guo et al. (2022) [42], Hempel et al. (2018) [43], Hong et al. (2021) [44], Kickingereder et al. (2015) [45], Lee et al. (2015) [46], Lee et al. (2019) [47], Lee_MH et al. (2019) [48], Lu et al. (2021) [49], Ozturk et al. (2021) [50], Prysiazhniuk et al. (2024) [52], Pruis et al. (2022) [51], Song et al. (2020) [53], Tan et al. (2016) (WHO II) [54], Tan et al. (2016) (WHO III) [54], Tan et al. (2016) (WHO IV) [54], Zhang et al. (2020) [55].

Finally, leave-one-out sensitivity analyses showed that the exclusion of individual studies did not substantially alter the global estimates for SMD (range: −0.897 to −0.789) or for AUC (range: 0.812 to 0.851), indicating stability in the results. However, heterogeneity (I^2^) decreased slightly when certain studies were excluded, particularly Guo et al. (2021) [42] for SMD and Lee et al. (2015) [46] for AUC, suggesting that they contributed more to the observed variability. In addition, the exclusion of studies with imputed data did not significantly alter the association between rCBV and IDH mutational status (SMD = −0.874 vs. −0.865; pooled AUC for studies with reported rCBV cutoff = 0.857 vs. 0.828) and led to a very slight decrease in heterogeneity in SMD (I^2^ = 59.2% vs. 62.8%) and a mild decrease in pooled AUC (I^2^ = 68.0% vs. 50.6%), suggesting that these studies did not significantly contribute to the observed variability. Figure 11 shows the results of the leave-one-out sensitivity analyses for SMD and AUC.

## 4. Discussion

This study focused on evaluating the diagnostic performance of mean rCBV obtained in DSC-MRI to differentiating between IDH-mutant- and IDH-wild-type brain gliomas. We included a total of 18 studies encompassing 1733 patients. We found that, despite moderate heterogeneity, the overall diagnostic accuracy of mean rCBV is high, with pooled sensitivity, specificity and AUC values of approximately 0.80. These results were consistent between different statistical approaches (pooled AUC, sensitivity and specificity, HSROC, and bivariate random-effects meta-analysis). Moreover, our analysis was further supported by a pooled DOR of 14.2, indicating a moderate-to-high discriminative power. In addition, meta-regression analyses did not reveal any significant influence of DSC-MRI acquisition parameters, although a trend toward significance was found for the flip angle. However, the relatively low number of included studies limit the reliability of meta-regression analyses. In sum, this meta-analysis not only supports the utility of mean rCBV to differentiate between IDH-mutant- vs. -wild-type gliomas but also quantifies its diagnostic performance through consistent and complimentary analyses.

Two previous meta-analyses explored the role of DSC-MRI perfusion metrics in predicting IDH mutation status. Van Santwijk et al. [56] conducted a meta-analysis including studies exploring T1-DCE and DSC MRI perfusion parameters in the differentiation of low- and high-grade glioma, as well as IDH mutation status. They included 12 studies with 1384 patients and found that CBV, ktrans, Ve, and Vp values were, in general, significantly higher in IDH-wild-type compared to IDH. The reported AUC for CBV values was 0.85 (95%-CI 0.75–0.93), but only three studies with DSC-MRI were included in their analysis. In addition, they included studies that reported CBV-related values not conventionally used in radiological practice, such as leakage (i.e., CBV-uncorrected—CBV corrected) [57], which represents a notable limitation.

Siakallis et al. [58] reviewed 16 studies including 1819 patients and analyzed a number of CBV-related metrics (e.g., mean rCBV, max rCBV, 75th percentile rCBV) and reported that the highest pooled specificity to differentiate between IDHm and IDHwt was observed for mean rCBV (82%), whereas rCBV 10th percentile showed maximum pooled-sensitivity, AUC, and DOR values (92%, 0.91, and 0 20.96, respectively). However, only two studies reported the latter measure, limiting its generalizability. In clinical practice, most software packages for image post-processing offer mean rCBV values, which seem more intuitive and stable compared to low and high percentiles, which may be affected by errors (e.g., inadequate tumor segmentation).

Our results align with those of the above-mentioned meta-analyses but focused on mean rCBV. Of note, all but one [49] of the included studies in our meta-analysis permitted a reliable estimation of mean rCBV. The study that only reported mean CBV values was only included in the SMD meta-analysis, since the statistical assumptions of this specific analysis allow for using mean CBV—not rCBV—to pool between-group differences. The pooled-sensitivity, specificity, and AUC values for the mean rCBV (80%) reinforce the validity of using mean rCBV as a biomarker for decision-making in clinical and radiological practice.

Nevertheless, the substantial variability found between the included studies calls for caution, since the range of reported AUCs for mean rCBV ranged from almost null (0.50 in Lee et al. (2015) [46]) to almost perfect (0.94 for Tan et al. (2016) [54] in WHO IV patients), and significant heterogeneity (I^2^ ranging from 62.8% to 91.8%) was observed. Such variability led us to explore possible modulating factors, particularly DSC-MRI acquisition parameters, which have been previously reported to influence mean rCBV measures. In fact, it is known that different MRI acquisition parameters may influence signal intensity of DSC-MRI perfusion, with subsequent variations in rCBV. For instance, Leu et al. (2017) showed that varying these parameters can impact the estimation of relative cerebral blood volume (rCBV) in gliomas, with different acquisition strategies yielding varying degrees of fidelity in CBV estimation [59]. Siakallis et al. [58] found that shorter TEs and smaller slice gaps were associated with higher sensitivities of mean rCBV.

Meta-regression analyses did not identify a dominant factor explaining this heterogeneity, precluding us from supporting Siakallis et al.’s findings in the specific context of mean rCBV, probably due to the low number of studies. However, we found statistical cues (*p* = 0.055) that lower flip-angle values are associated with smaller SMDs, suggesting that technical variability may contribute to differences in reported diagnostic performance. In fact, previous studies have found that a lower flip angle can reduce T1-weighting effects from contrast agent leakage, potentially yielding more accurate rCBV estimations in tumors with significant blood–brain barrier disruption [60,61]. Conversely, a higher flip angle may enhance signal-to-noise ratio (SNR), which, based on our results, seems to improve the discriminatory ability of mean rCBV—despite potential bias due to residual T1 effects. Notably, a balance between leakage contamination and SNR can be reached with different acquisition parameter configurations [62]. In sum, the observed trend toward significance in our meta-regression suggests that flip angle is a relevant parameter when standardizing DSC-MRI acquisition protocols for glioma assessment and, future studies should further investigate the impact of flip angle variations on rCBV reproducibility and diagnostic accuracy.

Be that as it may, the lack of standardization in parameter acquisition is evident in the included studies, and this underlying problem is further complicated considering the variability depending upon magnetic field [19], contrast agent doses [63], or imaging post-processing software [64]. In fact, another potential source of bias lies in the post-processing method used in each study, a problem that widely affects a number of neuroimaging-related clinical challenges, both in CT [65] and MRI. Up to seven different software packages were used in the included studies, the most frequent of which was NordicIce. Each of these packages include a specific pipeline and internal algorithms for parameter estimation. Moreover, each software tool may include a variety of post-processing algorithms. For instance, the NeuroPerfusion module of IntelliSpace Portal v. 11.0 (Phillips^®^) includes four different reconstruction algorithms, namely gamma-variate, model-free, manual AIF, and leakage correction [66]. Each of these reconstruction methods include mathematical nuances that result in different rCBV quantifications. The variability in rCBV based on different post-processing algorithms has also been demonstrated in the literature. For instance, Kudo et al. found that rCBV values of tumor and cutoff values for discriminating low- and high-grade gliomas differed between software packages, suggesting that optimal software-specific cutoff values should be used for diagnosis of high-grade gliomas [67].

Although limited by the relatively low number of included studies, the significant heterogeneity observed, along with existing knowledge on the influence of acquisition parameters (TE, TR, FA, etc.) and post-processing strategies, calls for the need of standardizing DSC-MRI parameter acquisition and harmonization of post-processing methodologies across different software platforms to improve the generalizability of rCBV as a biomarker for IDH mutation status. Multi-center prospective studies using uniform imaging and analysis pipelines will be essential to validate rCBV as a reproducible and clinically actionable biomarker. In sum, there is an emerging need for standardization not only in acquisition parameters but also in post-processing methods.

In the context of brain gliomas, significant efforts have been made to further extend the interpretability of rCBV in biological terms. An outstanding example is represented by studies exploring the so-called tumor vascular habitats, which are linked to different biological tumor features, as demonstrated by transcriptomic correlations [68,69,70]. For instance, Álvarez-Torres et al. applied complex clustering algorithms to delineate four vascular habitats within brain gliomas and peritumoral edema based on a mixture of rCBV and rCBF data. These vascular habitats are heuristically dichotomized into high and low angiogenic tumor habitats and have been found to significantly differentiate between IDH-wild-type glioblastoma and IDH-mutant astrocytoma [71].

In the current AI paradigm, a number of studies are increasingly exploiting the advantages of machine learning methods to extract information of perfusion MRI metrics that escapes conventional approaches [72]. We excluded these studies, as they are currently circumscribed to the research field, and the variability in methodological approaches precludes withdrawing generalizable conclusions. Notably, the studies by Kickingereder et al. (2015) [45] and Lee et al. (2019) [48] included in our meta-analysis applied machine learning algorithms to improve the diagnostic yield of perfusion MRI in diagnosing IDH mutation status as well as the 1-year overall survival in patients with brain glioma. They found that their developed ML model significantly outperformed the diagnostic yield of rCBV applied as per conventionally performed in clinical practice.

Similarly, radiomics has emerged as a novel methodology to extract meaningful quantitative features from imaging studies [73]. The application of radiomics to predict genomic data (i.e., radiogenomics) has shown promising results in the clinical problem approached in this study [74,75,76]. For instance, Bhandari et al. [77] found pooled-sensitivity and specificity values close to 90% in predicting IDH and 1p19q codeletion status of brain gliomas. In this context, interesting insights can be drawn from the combination between radiomics and perfusion MRI, since the latter provides temporal information that is absent in conventional MRI sequences [78,79,80]. Therefore, future meta-analysis should also explore the role of these recently developed approaches.

This study has several limitations: First of all, this meta-analysis aimed to focus on mean rCBV obtained in T2*-DSC perfusion MRI. This specific nature precludes us from withdrawing any conclusion regarding other MRI sequences (e.g., dynamic contrast enhancement, arterial spin labeling) or other rCBV metrics. Second, some studies did not report direct mean rCBV values, which implied the need to estimate them indirectly. Although the formulae applied for such conversion are widely accepted and used in the literature, estimation errors cannot be excluded. Third, although we did not detect significant publication bias, the presence of unpublished studies or studies reporting negative results could still influence the overall conclusions. Finally, some potentially relevant variables, such as the WHO glioma grade, were not considered in our analyses due to the heterogeneity in the edition of the WHO classification among the included studies, which varied from the 2007 edition [54] to the current 2021 edition [52]. These limitations should be overcome in future studies.

## 5. Conclusions

Mean rCBV is a reliable DSC-MRI parameter for differentiating between IDH-mutated- and IDH-wild-type brain gliomas, with significantly lower values in the former. Pooled sensitivity, specificity and AUC values of 80%, and DOR of 14.21 were observed. However, considerable heterogeneity in acquisition parameters, post-processing methods, and tumor segmentation limit optimal comparability between studies. Our results highlight the need for standardizing DSC-MRI perfusion to establish generalizable cutoff values of mean rCBV.

## Figures and Tables

**Table 1:** Main characteristics of the included studies. * No data provided for each subgroup, but all gliomas were “high-grade gliomas”. ^ Number of women with respect to the total sample of the study, which was larger than the analyzed patients (i.e., those with known IDH status).

Study (Year) [Reference]	Country	N	Age	Women	WHO-II	WHO-III	WHO-IV	IDH-M	IHD-wt
Ahn et al. (2023) [38]	Republic of Korea	132	46 ± 13	66	54	78	0	87	45
Brendle et al. (2020) [39]	Germany	56	48 ± 16	23	29	20	7	32	24
Choi et al. (2019) [40]	Republic of Korea	463	52.2 ± 14.8	191	32	142	289	125	338
Cindil et al. (2022) [41]	Turkey	58	49 ± 17 (IDHm); 58 ± 14 (IDHwt)	27	0	29 *	29 *	23	35
Guo et al. (2022) [42]	China	102	43.5 (18–74)	46	37	22	43	54	48
Hempel et al. (2018) [43]	Germany	100	51.4 ± 15.2	45	40	30	30	54	46
Hong et al. (2021) [44]	Republic of Korea	76	47.69 (19–68)	29	0	76	0	47	29
Kickingereder et al. (2015) [45]	Germany	73	43 ± 14	31	34	49	0	60	13
Lee et al. (2015) [46]	Republic of Korea	52	49.8 ± 14.5	20	0	36	16	16	36
Lee et al. (2019) [47]	Republic of Korea	110	47.44 ± 13.40	54	45	65	0	19	45
Lee_MH et al. (2019) [48]	Republic of Korea	88	52 (20–80)	41	0	0	88	12	76
Lu et al. (2021) [49]	China	71	53 (18.0–74.0)	36	0	0	71	45	26
Ozturk et al. (2021) [50]	USA	47	54 (20–90)	24	0	0	47	7	40
Pruis et al. (2022) [51]	The Netherlands	99	53.4 ± 15.3	36	78	17	4	81	18
Prysiazhniuk et al. (2024) [52]	Norway	66	47.07 ± 14.84	36 ^	19	13	34	33	33
Song et al. (2020) [53]	China	52	51.23 ± 15.59	21	16	6	30	22	30
Tan et al. (2016) (WHO II) [54]	China	31	38.94 ± 10.31 (IDHm); 51.57 ± 17.71 (IDHwt)	14	31	0	0	17	14
Tan et al. (2016) (WHO III) [54]	China	24	44.56 ± 43.33 (IDHm); 43.33 ± 13.85 (IDHwt)	10	0	24	0	9	15
Tan et al. (2016) (WHO IV) [54]	China	36	39.50 ± 10.10 (IDHm); 51.77 ± 13.57 (IDHwt)	12	0	0	36	6	30
Zhang et al. (2020) [55]	China	43	47 ± 13	23	14	14	15	20	23

**Table 2:** Meta-regression analysis results for standard mean difference. TRH, test for residual heterogeneity.

Variable	β Coefficient (95%CI)	*p*-Value	I^2^	Tau^2^	*p*-Value (TRH)
TE	−0.0011 (−0.0311, 0.0289)	0.9441	68.61%	0.1440	<0.0001
TR	−0.0004 (−0.0012, 0.0004)	0.345	67.71%	0.1349	<0.0001
FA (º)	−0.0091 (−0.0183, 0.0002)	0.0551	66.68%	0.1355	0.0002
Slice thickness	0.0301 (−0.3125, 0.3727)	0.8634	71.12%	0.1533	<0.0001
Slice gap	0.4497 (−0.1306, 1.0301)	0.1288	68.48%	0.1358	0.0007
N. images	−0.0352 (−0.1188, 0.0484)	0.4089	76.30%	0.2355	<0.0001
Scan time	0.0031 (−0.0124, 0.0187)	0.6922	78.27%	0.1880	<0.0001

**Table 3:** Meta-regression analysis results for area under the curve. TRH, test for residual heterogeneity.

Variable	β Coefficient (95%CI)	*p*-Value	I^2^	Tau^2^	*p*-Value (TRH)
TE	0.0009 (−0.0075, 0.0092)	0.8424	72.60%	0.0069	0.0007
TR	0.0001 (0.0002, 0.0004)	0.6953	72.37%	0.0069	0.0009
FA (º)	0 (−0.0048, 0.0047)	0.9856	44.42%	0.0021	0.1047
Slice thickness	0.0102 (−0.0796, 0.0999)	0.8241	70.49%	0.0071	0.0008
Slice gap	−0.0152 (−0.1863, 0.1559)	0.8620	74.03%	0.0092	0.0025
N. images	−0.0032 (−0.0307, 0.0244)	0.8213	84.19%	0.0164	0.0016
Scan time	0.0021 (−0.0059, 0.0101)	0.6099	50.46%	0.0036	0.1329

## Data Availability

All data are available as Supplementary Materials. For specific extracted information of the review process, data are available upon reasonable request to the corresponding author.

## Supplementary Materials

The supporting information can be downloaded at: https://www.mdpi.com/article/10.3390/diagnostics15070896/s1. Supplementary File S1 (Prisma Checklist); Supplementary Files S2–S4 (Search equations in PubMed, Web of Science and EMBASE, respectively); Supplementary Table S1 (Number of MRI scanners and post-processing information of the included studies); Supplementary Table S2 (Acquisition parameters according to the MRI scanners in each study); Supplementary Table S3 (QUADAS-2 Assessment by items).
