# Supplementary material for: The Value of Cerebral Blood Volume Derived from Dynamic Susceptibility Contrast Perfusion MRI in Predicting IDH Mutation Status of Brain Gliomas—A Systematic Review and Meta-Analysis
Source: Diagnostics (Basel). 2025 Apr 1;15(7):896. doi: 10.3390/diagnostics15070896 (PMC11989136; doi:10.3390/diagnostics15070896)
Supplement: Supplementary file 1 [file diagnostics-15-00896-s001.zip › Supplementary Table S1.pdf]

Study (year)	N. MRIs	DSC sequence pulse	Contrast dose/rate	Tumor segmentation	Post-processing software	AIF	Algorithm	Leakage correction (software or pre-bolus)	DSC Normalization
**Ahn et al. (2023)**	1	Gradient Echo-Echo Planar	NS	Semi-automatic + Manual	NordicICE	NS	NS	Yes	Normalized
**Brendle et al. (2020)**	1	Gradient Echo-Echo Planar	0.1/3	Automatic with NR supervision	Syngo	Manual	Model-Based leakage correction	Yes	Normalized
**Choi et al. (2021)**	5	NS	0.1/NS	Automatic	NordicICE	Automatic	NS	No	Standardized [temporal + feature-wise normalization]
**Cindil et al. (2021)**	1	Fast-Echo-Planar T2-weighted Gradient Echo	0.1/5	Manual	NS	NS	NS	No	Normalized
**Guo et al. (2021)**	1	Fast Field Echo- Echo-Planar imaging	0.2/3.5	Manual	Func- Tool	NS	NS	NS	Normalized
**Hempel et al. (2019)**	1	Gradient Echo-Echo Planar	2x0.1/3	Manual	Syngo	Manual	NS	Yes	Normalized
**Kickingereder et al. (2015)**	1	Gradient Echo-Echo Planar	0.1/NS	Automatic	Olea Sphere	Automatic	Boxerman et al.	Yes	Standardized [Spaced clusters]
**Hong et al. (2021)**	6	NS	NS	Manual	NordicICE	NS	NS	Yes	Normalized
**Lee et al. (2015)**	1	Gradient Echo-Echo Planar	0.1/4	Manual	NordicICE	NS	NS	Yes	Normalized
**Lee et al. (2019)**	1	Gradient Echo-Echo Planar	0.1/4	Manual	NordicICE	NS	Boxerman-Weiskoff	Yes	Normalized
**Lee_MH et al. (2019)**	1	Gradient Recalled T2*-weighted Echo-Planar	NS	Manual	NordicICE	NS	Gamma variate for recirculation. Leakage	Yes	Standardized

							corrected but method NS		
**Lu et al. (2021)**	1	NS	0.1/4	Manual	AW	NS	Gamma variate	Yes	No
**Ozturk et al. (2021)**	1	Gradient Echo-Echo Planar	0.1/5	Manual	DynaSuite	NS	Boxerman-Weiskoff	Yes	Normalized
**Prysiazhniuk et al. (2024)**	1	Gradient recalled EPI	0.1/5	Automatic	NordicICE	Automatic	NS	Yes	Normalized
**Pruis et al. (2022)**	3	NS	NS/4-5	Semi-automatic	OsiriX-plugin IB-Neuro	Manual	NS	Yes	Standardized [Voxel Intensity Standardized signal values]
**Song et al. (2021)**	1	Gradient Recalled T2*-weighted Echo-Planar	0.1/4-5	Manual	AW	Automatic	NS	Yes	Normalized
**Tan et al. (2016)**	1	Gradient Echo-Echo Planar	NA/3.5	Manual	Syngo	Manual	NS	NS	Normalized
**Zhang et al. (2020)**	1	Gradient Echo-Echo Planar	0.1/3.5	Manual	Syngo	Manual	NS	NS	Normalized

Supplementary Table S1. Number of MRI scanners and post-processing information of the included studies. AIF, Arterial Input Function. Syngo, Syngo® MR Perfusion (Siemens Healthineers, Erlangen Germany). NordicICE, NordicICE, NordicNeuroLab, Inc. Milwaukee, WI, USA. Func-Tool (GE Healthcare). Olea Sphere, Olea Sphere (Olea Medical, La Ciotat, France). AW, AW Workstation (GE Healthcare). NR, neuroradiologist*NS. Not specified.
