# Supplementary material for: The Value of Cerebral Blood Volume Derived from Dynamic Susceptibility Contrast Perfusion MRI in Predicting IDH Mutation Status of Brain Gliomas—A Systematic Review and Meta-Analysis
Source: Diagnostics (Basel). 2025 Apr 1;15(7):896. doi: 10.3390/diagnostics15070896 (PMC11989136; doi:10.3390/diagnostics15070896)
Supplement: Supplementary file 1 [file diagnostics-15-00896-s001.zip › Supplementary Table S2.pdf]

Study (year)	N	TR (ms)	TE (ms)	FA (°)	NEX	Matrix	ST	SG	FOV	Scan time (s)	N. of images
**Ahn et al. (2023)**	132	1600	30	40	NS	256x256	5	NS	220x240	NS	60
**Brendle et al. (2020)**	56	1130	31	60	NS	128x128	4	NS	230x230	NS	NS
**Choi et al. (20219) (SIGNA EXCITE. GE)**	93	1500	40	35	1	128x128	5	1	240x240	90	NS
**Choi et al. (2019) (SIGNA SIGNA HDXT. GE)**	93	1500	40	35	1	128x128	5	1	240x240	90	NS
**Choi et al. (2019) (DISCOVERY MR750W. GE)**	93	1500	29.3	60	1	100x100	5	1.5	240x240	90	NS
**Choi et al. (2019) (VERIO. SIEMENS)**	92	1600	30	90	1	128x128	6	0.9	240x240	104	NS
**Choi et al. (2019) (SKYRA. SIEMENS)**	92	1600	30	90	1	128x128	6	0.9	240x240	104	NS
**Cindil et al. (2021)**	58	1500	30	90	NS	128x128	5	1.5	230x230	82.8	60
**Guo et al. (2021)**	102	1700	40	75	NS	NS	6	0	NS	107	60
**Hempel et al. (2019)**	100	1130	31	NS	NS	128x128	4	NS	230x230	NS	NS
**Kickingereder et al. (2015)**	73	2220	36	90	NS	128x128	5	NS	240x240	NS	50-75
**Hong et al. (2021) (SIGNA EXCITE. GE)**	2	1500	40	35	1	128x128	5	1	220x220	90	NS
**Hong et al. (2021) (SIGNA HDXT. GE)**	16	1500	40	35	1	128x128	5	1	240x240	90	NS
**Hong et al. (2021)(DISCOVERY MR750W. GE)**	4	1500	29	60	1	100x100	5	1.5	240x240	90	NS
**Hong et al. (2021) (VERIO. SIEMENS)**	46	1600	30	90	1	128x128	6	0.9	240x240	104	NS
**Hong et al. (2021) (SKYRA. SIEMENS)**	5	1600	30	90	1	128x128	6	0.9	240x240	104	NS
**Hong et al. (2021) (TrioTIM. SIEMENS)**	3	1500	30	90	1	128x128	5	1	240x240	104	NS
**Lee et al. (2015)**	52	1500	35	NS	NS	128x128	5	1	240x240	NS	60
**Lee et al. (2019)**	110	1808	40	35	NS	128x128	5	2	240x240	154	NS
**Lee_MH et al. (2019)**	88	1720	35	40	NS	128x128	5	NS	NS	90	50
**Lu et al. (2021)**	71	1500	30	NS	NS	128x128	4	1.2	220x220	78	60
**Ozturk et al. (2021)**	47	1500	43	NS	1	NS	NS	NS	230x230	NS	NS
**Prysiazhniuk et al. (2024)**	66	1875	45	60	NS	128x128	5	1	240x240	84	53
**Pruis et al. (2022) (Erasmus MC. GE)**	76	2000	45	90	NS	128x128	5-6	NS	NS	NS	NS
**Pruis et al. (2022) (HMC. SIEMENS)**	14	2400	46	70	NS	256x224	5-6	NS	NS	NS	NS
**Pruis et al. (2022) (HMC2. SIEMENS)**	9	1490	30	90	NS	128x128	5-6	NS	NS	NS	NS
**Song et al. (2021)**	52	1800	15.4	90	1	NS	5	1	240x240	90	50
**Tan et al. (2016)**	31	1500	30	90	NS	128x128	4	1.2	230x230	NS	NS
**Zhang et al. (2020)**	43	1600	30	90	1	128x128	4	NS	220x220	104	60

Supplementary Table S2. Acquisition parameters according to the MRI scanners in each study. N/MRI, number of patients. TR, Repetition Time. TE, Echo Time. FA, Flip Angle. NEX, Number of Excitations. ST, Slice Thickness (mm). SG, Slice Gap (mm). FOV, Field Of View (mm x mm).*NS. Not specified.
