# Supplementary material for: The Value of Cerebral Blood Volume Derived from Dynamic Susceptibility Contrast Perfusion MRI in Predicting IDH Mutation Status of Brain Gliomas—A Systematic Review and Meta-Analysis
Source: Diagnostics (Basel). 2025 Apr 1;15(7):896. doi: 10.3390/diagnostics15070896 (PMC11989136; doi:10.3390/diagnostics15070896)
Supplement: Supplementary file 1 [file diagnostics-15-00896-s001.zip › Supplementary Table S3.pdf]

	1	2	3	4	5	6	7	8	9	10	11	12	13	14	15	16	17	18	19
Ahn et al. (2023)	Unclear	Yes	Unclear	Unclear	Low	rCBV	Unclear	Unclear	Low	IDH	Yes	Unclear	Low	Low	Unclear	Yes	Yes	Yes	Low
Brendle et al. (2020)	Yes	Yes	Unclear	Unclear	Low	rCBV	Unclear	Unclear	Low	IDH	Yes	Unclear	Low	Low	Unclear	Yes	Yes	Yes	Low
Choi et al. (2019)	Yes	Yes	Yes	Low	Low	rCBV	Unclear	Unclear	Low	IDH	Yes	Unclear	Low	Low	Unclear	Yes	Yes	Yes	Low
Cindil et al. (2021)	Unclear	Yes	Unclear	Unclear	Low	rCBV	Yes	Low	Low	IDH	Yes	Unclear	Low	Low	Yes	Yes	Yes	Yes	Low
Guo et al. (2021)	Unclear	Yes	No	Unclear	Low	rCBV	Unclear	Unclear	Low	IDH	Yes	Yes	Low	Low	Unclear	Yes	Yes	Yes	Low
Hempel et al. (2019)	Yes	Yes	Yes	Low	Low	rCBV	Yes	Low	Low	IDH	Yes	Unclear	Low	Low	Yes	Yes	Yes	Yes	Low
Kickingereder et al. (2015)	Yes	Yes	Yes	Low	Low	rCBV	Unclear	Unclear	Low	IDH	Yes	Unclear	Low	Low	Unclear	Yes	Yes	Yes	Low
Hong et al. (2021)	Unclear	Yes	Yes	Low	Low	rCBV	Unclear	Unclear	Low	IDH	Yes	Unclear	Low	Low	Unclear	Yes	Yes	Yes	Low
Lee et al. (2015)	Yes	Yes	Low	Low	Low	rCBV	Unclear	Unclear	Low	IDH	Yes	Unclear	Low	Low	Unclear	Yes	Yes	Yes	Low
Lee et al. (2019)	Yes	Yes	Unclear	Unclear	Low	rCBV	Unclear	Low	Low	IDH	Yes	Unclear	Low	Low	Unclear	Yes	Yes	Yes	Low
Lee MH et al. (2019)	Unclear	Yes	Unclear	Unclear	Low	rCBV	Yes	Low	Low	IDH	Yes	Unclear	Low	Low	Unclear	Yes	Yes	Yes	Low
Lu et al. (2021)	Yes	Yes	Yes	Low	Low	CBV	Unclear	High	Unclear	IDH	Yes	Unclear	Low	Low	Unclear	Yes	Yes	Yes	Low
Ozturk et al. (2021)	Unclear	Yes	Yes	Low	Low	rCBV	Yes	Low	Low	IDH	Yes	No	Low	Low	Unclear	Yes	Yes	Yes	Low
Prysiazhniuk et al. (2024)	Unclear	Yes	Unclear	Unclear	Low	rCBV	Unclear	Unclear	Unclear	IDH	Yes	Unclear	Low	Low	Unclear	Yes	Yes	Yes	Low
Pruis et al. (2022)	Yes	Yes	Yes	Low	Low	rCBV	Unclear	Unclear	Unclear	IDH	Yes	Unclear	Low	Low	Unclear	Yes	Yes	Yes	Low
Song et al. (2021)	Yes	Yes	No	Unclear	Low	rCBV	Unclear	Unclear	Low	IDH	Yes	Unclear	Low	Low	Unclear	Yes	Yes	Yes	Low
Tan et al. (2016)	Yes	Yes	Unclear	Unclear	Low	rCBV	Yes	Low	Low	IDH	Yes	Unclear	Low	Low	Unclear	Yes	Yes	Yes	Low
Zhang 2020	Unclear	Yes	No	Unclear	Low	rCBV	Unclear	Unclear	Low	IDH	Yes	Unclear	Low	Low	Unclear	Yes	Yes	Yes	Low

Supplementary Table S3. QUADAS-2 Assessment. Numbers in the first row correspond to the following items of QUADAS-2: 1) Was a consecutive or random sample of patients enrolled? 2) Was a case-control design avoided? 3) Did the study avoid inappropriate exclusions? 4) Could the selection of patients have introduced bias? 5) Are there concerns that the included patients do not match the review question? 6) Describe the index test and how it was conducted and interpreted 7) Were the index test results interpreted without knowledge of the results of the reference standard? 8) Could the conduct or interpretation of the index test have introduced bias? 9) Is there concern that the index test, its conduct, or interpretation differ from the review question? 10) Describe the reference standard and how it was conducted and interpreted 11) Is the reference standard likely to correctly classify the target condition? 12) Were the reference standard results interpreted without knowledge of the results of the index test? 13) Could the reference standard, its conduct or its interpretation have introduced bias? 14) Are there concerns that the target condition as defined by the reference standard does not match the review question? 15) Was there an appropriate interval between index test(s) and reference standard? 16) Did all patients receive a reference standard? 17) Did all patients receive the same reference standard? 18) Were all patients included in the analysis? 19) Could the patient flow have introduced bias?
